# Revolutionizing Skin Cancer Triage: The Role of Patient-Initiated Teledermoscopy in Remote Diagnosis

**DOI:** 10.3390/cancers16142565

**Published:** 2024-07-17

**Authors:** Emilie A. Foltz, Joanna Ludzik, Sancy Leachman, Elizabeth Stoos, Teri Greiling, Noelle Teske, Lara Clayton, Alyssa L. Becker, Alexander Witkowski

**Affiliations:** 1 Department of Dermatology, Oregon Health & Science University, Portland, OR 97239, USA; 2 Elson S. Floyd College of Medicine, Washington State University, Spokane, WA 99202, USA; 3 Knight Cancer Research Institute, Oregon Health & Science University, Portland, OR 97239, USA; 4 John A. Burns School of Medicine, University of Hawai’i at Manoa, Honolulu, HI 96822, USA

**Keywords:** teledermatology, dermoscopy, teledermoscopy, melanoma triage, skin cancer triage, dermatology access, telehealth, telemedicine, early detection

## Abstract

**Simple Summary:**

This research investigates the use of teledermoscopy, a method that involves patients using a smartphone dermatoscope attachment to take pictures of skin lesions for remote evaluation by dermatologists. This approach aims to reduce the need for in-person visits for skin cancer screening and triage. This study found that incorporating smartphone dermatoscope images significantly reduced the number of lesions requiring in-person evaluation compared to clinical images alone. This suggests that patient-led teledermoscopy could decrease unnecessary visits for benign lesions, potentially improving access to dermatology consultations for patients with suspicious or malignant lesions. Overall, this research highlights a promising strategy to enhance skin cancer detection and streamline healthcare delivery in dermatology through telemedicine.

**Abstract:**

Introduction: Teledermatology, defined as the use of remote imaging technologies to provide dermatologic healthcare services to individuals in a distant setting, has grown considerably in popularity since its widespread implementation during the COVID-19 pandemic. Teledermoscopy employs a smartphone dermatoscope attachment paired with a smartphone camera to visualize colors and microstructures within the epidermis and superficial dermis that cannot be seen with the naked eye ABCD criteria alone. Methods: Our retrospective observational cohort and case–control study evaluated the utility of loaning a smartphone dermatoscope attachment to patients for remote triage of self-selected lesions of concern for skin cancer. The primary outcome was the number (percentage) of in-person follow-up visits required for patients who submitted lesion images, either with or without accompanying dermoscopic images. A medical record review was conducted on all Oregon Health & Science University Department of Dermatology spot check image submissions utilizing the smartphone dermatoscopes between August 2020 and August 2022. De-identified dermoscopic images of lesions that included corresponding non-dermoscopic clinical images in their submission (n = 70) were independently reviewed by a blinded expert dermoscopist. The expert used standard clinical algorithms (ABCD criteria for clinical images; dermoscopy three-point checklist for dermoscopic images) to determine whether the imaged lesion should be converted to an in-person visit for further evaluation and consideration for biopsy. Results: Of the 70 lesions submitted with corresponding clinical and dermoscopy images, 60 met the criteria for in-person evaluation from clinical (non-dermoscopic) image review compared to 28 meeting the criteria for in-person evaluation from dermoscopic images of the same lesion. Thus, a 53% reduction in conversion to an in-person consultation with the addition of smartphone dermatoscope images in virtual lesion triage was observed (*p* < 0.001, McNemar’s Test). Conclusion: Implementing patient-led teledermoscopy may reduce the frequency of in-person visits for benign lesions and consequently improve access to in-person dermatology consultations for patients with concerning and possibly malignant lesions.

## 1. Introduction

In recent years, a significant shortage of primary care physicians and dermatologists has resulted in prolonged wait times and decreased access to dermatologists for patients with skin neoplasms of concern [1]. This issue is a growing problem in the healthcare system, causing delays in diagnosis and treatment. The limited availability of these specialists means that patients often have to wait months for an appointment, during which time their condition could worsen. The situation has been further exacerbated by the COVID-19 pandemic, which introduced several additional factors that have compounded the problem. These factors include a reduced desire for in-person consultations due to the risk of exposure to the virus, increased financial restrictions limiting the ability to commute to office visits, sickness that requires quarantine, and geographically limited access to healthcare. Together, these challenges have created a perfect storm that has increased the utilization of teledermatology for the secondary prevention of melanoma and nonmelanoma skin cancer [2,3]. The shift toward telemedicine has been necessary to continue providing care while minimizing the risk of virus transmission.

In this context, a greater responsibility is being placed on patients to perform self-skin examinations (SSEs) and evaluate their own skin lesions of concern. Unfortunately, patient-conducted SSEs can be challenging to implement effectively due to inadequate education regarding skin cancer risk factors and appearance, as well as poor mechanisms for recalling skin changes [4]. Many patients are not familiar with the various risk factors associated with skin cancer, nor are they trained to recognize the appearance of suspicious skin lesions. This lack of education and training can lead to missed or delayed diagnoses, further complicating patient outcomes. Furthermore, patients may have difficulty remembering the details of their skin changes over time, which is crucial for early detection and intervention. This highlights the need for better educational resources and support tools to help patients conduct SSEs more effectively and accurately.

Teledermoscopy, which utilizes smartphone attachments to visualize architectural patterns in the examination of skin lesions, has been explored in dermatology, primary care, and at-home settings to improve the virtual triage of suspicious skin lesions [5,6,7,8,9,10,11]. Teledermoscopy can be delivered through a virtual visit communication in real-time or through a store-and-forward (SAF) mechanism, which allows patients or providers to take digital dermoscopic images (DDIs) and submit them through a secure portal to a reviewing dermatology provider. This technology enables dermatologists to assess the images and provide feedback without the need for an in-person visit. When combined with real-time or SAF technology, teledermoscopy can significantly improve virtual referral and triage workflow [12,13,14,15]. This streamlined approach helps prioritize patients who need urgent care while managing those who can be monitored remotely. Moreover, several studies have indicated that both physicians and patients find the use of teledermoscopy favorable [16,17,18,19,20]. The ability to capture and share high-quality images of skin lesions without the need for an in-person visit is a significant advancement in the field of dermatology. This not only reduces the burden on healthcare facilities but also provides patients with timely access to specialist opinions.

The recent literature has examined the utilization of smartphone dermatoscopes to aid in communication between primary care providers and dermatology providers when assessing skin lesions of concern [21,22,23,24,25]. These studies highlight the potential for smartphone dermatoscopes to facilitate more accurate and timely assessments, bridging the gap between primary care and specialized dermatological care. Smartphone dermatoscopes can be easily used by primary care providers to capture detailed images of skin lesions, which can then be reviewed by dermatologists to determine the appropriate course of action. However, there is a need to more extensively explore the utility of smartphone DDIs sent by patients to their dermatology provider during SAF virtual spot checks. Understanding how patients can effectively use this technology at home is crucial for its broader adoption and efficacy. Ensuring that patients are comfortable and proficient in using these tools will be key to maximizing their benefits.

The primary objective of our study is to observe if the implementation of at-home teledermoscopy reduces the number of SAF spot checks that convert to in-person consultations by dermatologists. By analyzing these data, we aim to determine whether teledermoscopy can effectively triage patients and reduce the burden on dermatology clinics. A successful reduction in in-person consultations would indicate that teledermoscopy can be a viable alternative for managing certain skin conditions remotely. Our secondary objective is to observe trends within our cohort of participants employing the smartphone dermatoscope attachment in at-home SSEs. This includes examining how patients use the technology, their satisfaction with the process, and the accuracy of their self-assessments. By understanding these trends, we can identify potential areas for improvement and ensure that the technology is user-friendly and effective in helping patients manage their skin health.

## 2. Materials and Methods

### 2.1. Study Design

We evaluated the utilization of a smartphone dermatoscope attachment loaner program for patient-selected skin lesions of concern submitted via “e-visit” for a virtual spot check to an Oregon Health & Science University (OHSU) dermatology provider. The institutional review board at OHSU approved this retrospective observational cohort and case–control study (STUDY00018408) and waived the documentation of informed consent.

**Figure 1:**
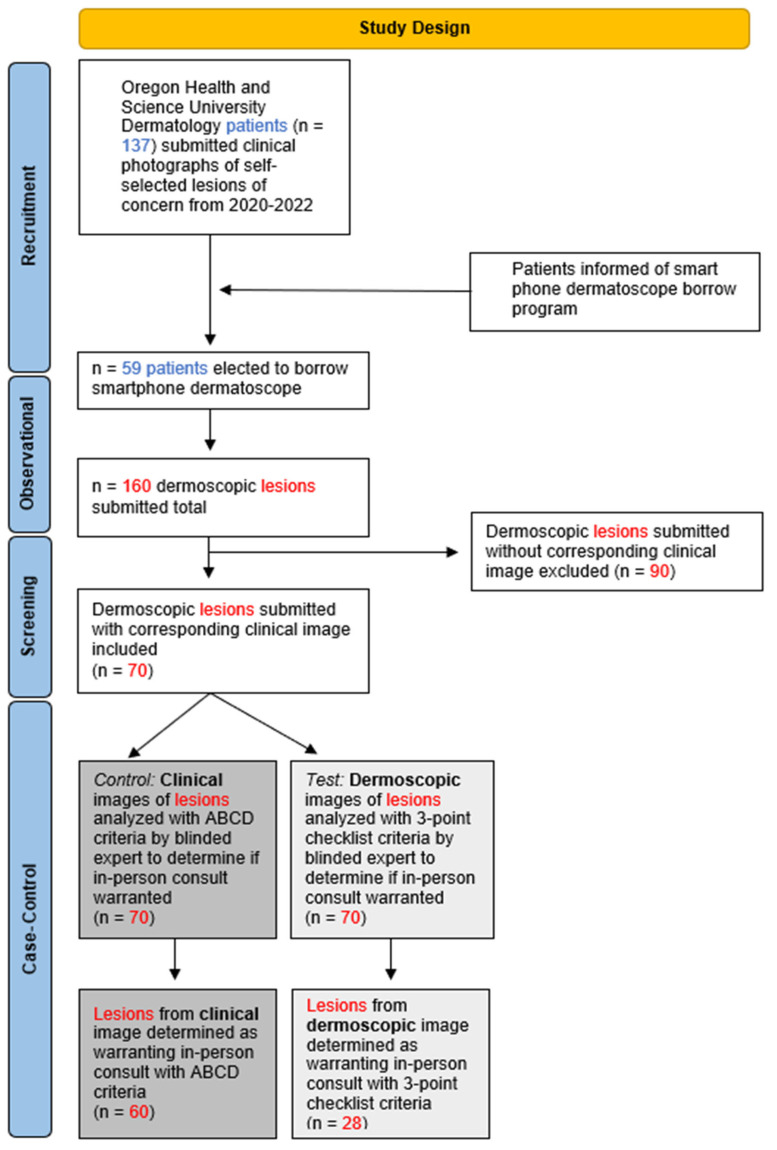
Overview of study design and primary outcomes.

The state of Oregon community was informed via the War on Melanoma^TM^ campaign [26] and OHSU Department of Dermatology patients were informed via patient portal communication about the opportunity to obtain a smartphone dermatoscope attachment (Sklip dermatoscope, Sklip Inc., Lake Oswego, OR, USA) through a loaner program (Sklip Loaner Program, Oregon Health & Science Department of Dermatology, Portland, OR, USA). Interested individuals were asked to complete an online form to request to participate in the program and were given training on the utilization of the device in video and written formats [27,28]. Requests for the device were fulfilled via a shipping platform (Shopify, Ottawa, ON, Canada). Devices were shipped within an average of 24 h and delivered in an average of 3 days via the United States Postal Service (USPS). Participants utilized the smartphone dermatoscope attachment to photograph self-selected skin lesions of concern and send them to their provider via a SAF spot check, which allowed for a remote dermatology provider to review and further communicate through their secure electronic medical record. All patients were asked to take one clinical image and one digital dermoscopic image (DDI) of their self-selected lesion(s) of concern. Submitted lesions lacking clinical and/or DDI were excluded from analysis in this study (Figure 1).

### 2.2. Measures and Statistics

In the case–control portion of this study, where a case was defined as an image without dermoscopy and the control was defined as dermoscopy applied to an image, a blinded dermoscopy expert triaged the imaged skin lesion into one of two categories: no concern with follow-up as needed or warranting conversion into an in-person dermatology visit for consideration to biopsy. This determination was based on the following criteria for clinical images and DDIs: ABCD criteria (asymmetry, irregular border, multiple colors, border ≥ 6 mm) [29,30,31] was applied to clinical images, and the dermoscopy 3-point checklist (D3PC) (irregular network, asymmetry, blue-white structures) [32] was applied to DDIs. A binary system was utilized to assess whether each individual image submission met the aforementioned criteria for conversion to an in-person visit, where 1 = criteria met and 0 = criteria not met. Clinical images and DDI were reviewed on separate days and in a randomized and de-identified format by the blinded expert; however, each lesion in the dermoscopy cohort had a clinical image for every corresponding dermoscopic image. A McNemar’s Chi Squared test was conducted to quantify the statistical significance of the difference in the number of virtual spot check images meeting the criteria to convert to an in-person consultation.

An opportunity cost estimation was calculated for the average patient visiting OHSU Dermatology for an in-person visit. This was used to estimate the average expenditures saved by those not requiring an in-person consultation. The cost of an average office visit at OHSU Dermatology for a Medicare patient was considered. Additionally, the average cost savings in gas mileage for participants to stay home and participate in remote lesion triage, relative to converting to an in-person visit, was calculated. The distance in miles for each participant was measured from their home address to OHSU Dermatology, all distances were averaged, and the average miles per gallon for an Oregon state driver was cited. Subsequently, the average cost per gallon from 2020 to 2022 was calculated. Furthermore, the opportunity cost estimation considered wages lost, as influenced by the Oregon state minimum wage from 2020 to 2022 and the average total time spent commuting and attending a medical office visit.

Additional qualitative exploratory metrics were observed including patient demographics, the number of lesions submitted per participant, anatomic locations of lesions, dermoscopy-suggested diagnoses, and pathology results as applicable.

## 3. Results

Within a 2-year period (August 2020–August 2022), a total of 137 self-selected patients submitted clinical images of self-selected spots of concern, and 59 of those patients submitted DDIs using the smartphone dermatoscope. Patients included both new and established patients at OHSU. A total of 160 lesions were submitted for review with a dermoscopic image. On average, participants submitted 2.71 lesions (median: 1, range: 1–34) per virtual dermoscopy spot check (Table 1). The population of participating patients was 73% female-identifying (n = 43) and 27% male-identifying (n = 16). Of the 59 participants, 5% had devices shipped to rural counties (n = 3).

Of the 160 lesions photographed with the smartphone dermatoscopes, 15 were determined to require biopsy. Due to insufficient tissue collected, pathology was unable to assess two of the biopsied lesions, and as a consequence, a histopathologic result was not recorded. Of the remaining 13 biopsies, 3 yielded a malignant diagnosis. One was histologically determined as melanoma in situ (MIS) and the other two were histologically determined as basal cell carcinoma (BCC).

Of the 70 lesion submissions eligible for the case–control portion of this study, 60 warranted in-person evaluation from clinical image review compared to 28 warranting in-person evaluation from DDI review of the same lesion (Table 2). Thus, this is a 53% reduction in conversion to an in-person consultation with the addition of smartphone dermatoscope images in virtual lesion triage (*p* < 0.001, McNemar’s Test).

It was calculated that a minimum of USD 67.78 per person was saved to include travel, office visit costs, and wages lost to time off (Table 3). That totals USD 2168.96 saved among the 32 fewer participants requiring an in-person consultation with the application of teledermoscopy in their virtual spot checks. These calculations do not include additional expenditures associated with the office visit, such as costs for biopsies and specimen handling.

## 4. Discussion

Previous studies have reinforced the feasibility of teledermoscopy, achieving similar diagnostic and management accuracy as traditional in-office dermatology evaluations [37,38,39,40,41]. These studies highlight the potential of teledermoscopy to revolutionize the way dermatological care is delivered, especially in settings where access to in-person consultations is limited. The ability to remotely diagnose and manage skin conditions with a high degree of accuracy is a significant advancement in the field of dermatology, offering new avenues for patient care and management.

Image quality plays a crucial role in the accurate triage of concerning skin lesions by physicians in remote settings. Studies, such as Argenziano et al. (2006), have demonstrated that dermoscopy significantly enhances sensitivity and negative predictive value in diagnosing suspicious lesions compared to naked-eye evaluation (*p* < 0.005) [42]. This improvement in diagnostic accuracy is vital for remote consultations where visual clarity on the first attempt to evaluate a skin lesion of concern is paramount. High-quality images allow dermatologists to make more informed decisions, reducing the likelihood of missed or incorrect diagnoses. Ensuring that patients can capture and transmit high-resolution images is essential for the success of teledermoscopy programs.

With the application of teledermoscopy in remote lesion triage, it must be considered how patients can implement these mobile devices. This study can inform future studies, showcasing the average number of skin lesions examined in a remote SSE for spot check triage. Furthermore, we can deduce that the imbalance of participants in each gender suggests increased engagement of females with the program. Future studies should address the origin of this discrepancy. Understanding the factors that influence patient engagement and utilization of teledermoscopy tools is crucial for designing programs that are accessible and effective for all demographic groups. Efforts should be made to identify and overcome barriers that may prevent certain populations from participating fully.

A reduction in in-person consultations can save a health system and individuals substantial expenditures. This includes the patient often losing wages from work absence and resources being consumed in the hospital. Importantly, the 53% reduction in face-to-face consultations was statistically significant (*p* < 0.001). Allowing patients to remain at home for mobile spot check triage relieves a considerable financial burden (Table 3) and expands access to those who reside in medically underserved communities. The cost savings extend beyond the immediate healthcare system, benefiting patients who may face financial and logistical challenges associated with traveling to and from medical appointments. Additionally, reducing the demand for in-person consultations can help allocate resources more efficiently within the healthcare system, potentially improving overall patient outcomes.

There is currently a shortage of dermatologists in the United States [1]. Reducing the number of individuals requiring an in-person office visit could shorten the wait time for an in-person evaluation of suspicious skin lesions and time to biopsy and reduce face-to-face visit conversions for benign lesions. This may have implications for improved early detection of melanoma and nonmelanoma skin cancers. By streamlining the triage process and prioritizing cases that truly require in-person attention, teledermoscopy can help alleviate the burden on dermatology clinics, allowing them to focus on patients with the most urgent needs. This could lead to more timely interventions and better health outcomes for patients with serious skin conditions.

### Limitations

The external validity of this study is limited due to its sample size. The reasons for limited participation likely involved a lack of awareness of the novel loaner program and how to partake. Other reasons may have included a disinterest in engaging with dermoscopy training, as well as discrepancies in generational utilization and comfort of smartphone technologies.

The authors also acknowledge the concern for missing important indications of skin cancer at earlier stages through a reduction in in-person consultations. This could result in missed opportunities for timely treatment and may lead to misdiagnosis and aggravation of cancer in affected patients. While our study demonstrates the potential of at-home teledermoscopy to maximize diagnostic sensitivity by reducing false positive concerning lesions, it is still crucial for patients and healthcare providers to remain vigilant and consider follow-up consultations in cases of uncertainty or persistent concern. Given the retrospective nature of our case–control study, the results of our findings had no direct impact on patient care. Rather, our findings should be used to inform future study directions for further exploration into applicability to patient care.

Future studies should include a diverse array of clinicians from multiple continents for assessment of the images and should score them in a blinded way. Additionally, they should implement a larger sample size to ensure improved generalizability and continue to promote technology literacy in an equitable fashion to all populations. Addressing these limitations is crucial for enhancing the reliability and applicability of the findings.

Efforts should be made to increase awareness of teledermoscopy programs, providing clear instructions and support to encourage participation across diverse demographic groups. Additionally, understanding the barriers to engagement with smartphone technologies can help tailor educational initiatives to improve technology literacy and comfort among all patients, regardless of age or technological proficiency.

## 5. Conclusions

Our study allowed for the analysis of patient-implemented teledermoscopy for triage of self-selected skin lesions of concern. We were able to analyze the impact of this service by providing a loaner program to individuals who were patients at Oregon Health & Science University and by conducting a retrospective observational cohort and case–control study of the data.

Our findings suggest that patient-implemented teledermoscopy considerably reduced the number of spot checks that required conversion to an in-person consultation. This may ultimately reduce the frequency of in-person visits for benign lesions, and thus, may decrease wait times for other patients with concerning and possibly malignant lesions. Decreasing the frequency of unnecessary visits may not only improve patient quality of life but also promote cost-effective expenditures for health systems at large.

Providing smartphone dermatoscope attachments to all OHSU Dermatology patients serves as an opportunity to reduce in-person visits for benign lesions through enhanced visualization of lesions of concern. The ability to provide these devices to all individuals equitably is, to our knowledge, unique to our institution.

## Figures and Tables

**Table 1:** Findings of the observational portion of the study.

Sample Characteristics					
**Total number of participants submitting DDI**					n = 59
**Participant age (years)**					
	Mean				46
	Median				42
	≥65 years				n = 13
	≤64 years				n = 46
**Participant gender identity**					
	Male				27% (n = 16)
	Female				73% (n = 43)
Participant distance from home to dermatology clinic * (miles)					
	Mean				22.6
	Median				8.8
**Total number of lesions submitted with DDI**					n = 160
**DDI of individual lesion(s) submitted per participant**					
	1 lesion 2 lesions 3 lesions 4 lesions 5 lesions 6 lesions >10 lesions Mean				n = 35 n = 11 n = 6 n = 1 n = 1 n = 1 n = 4 2.71
	Median				1
**Lesions biopsied**					9% (n = 15)
	Malignant Benign Insufficient tissue for analysis				20% (n = 3) 67% (n = 10) 13% (n = 2)
DDI resulting in in-person consultation **					**18% (n = 29)**

* OHSU Dermatology; ** Real world outcomes from loaner program.

**Table 2:** Outcomes from retrospective case-control portion of study.

Retrospective Case-Control Outcomes		
Total number of participants who submitted both DDI and clinical image		n = 46
Total number of lesions submitted with both DDI and clinical image		n = 70
Lesions meeting ABCD criteria from clinical image		86% (n = 60)
Lesions meeting D3PC criteria from DDI		40% (n = 28)

**Table 3:** Estimated average expenditures saved by not visiting dermatology clinic in-person.

Opportunity Costs for the Average Patient in Oregon State visiting OHSU Dermatology	
Cost of one visit for average patient with Medicare *	36.72 USD
Total gas expenditure per patient ((19.41 × 2) × 3.68)/25.7)	5.56 USD
*Average distance from sample population to dermatology clinic* **	*19.41 miles*
*Cost of gas per gallon, average between 2020 and 2022* [33]	*3.68 USD*
*Average miles per gallon for 2020 vehicle* [34]	*25.7 miles*
Total wages lost for time spent (12.75 × 2)	25.50 USD
*Minimum wage, Oregon state* ^‡^ [35]	*12.75 USD/hour*
*Average total time spent per medical office visit* [36]	*2 h*
**Total opportunity cost estimated on average**	**$67.78**

* Cost based on relative value units of a standard office visit for an established patient at OHSU Dermatology. ** Distance from participant-provided home address to OHSU dermatology, averaged among participants. ^‡^ Averaged between 2020 and 2022.

## Data Availability

The data presented in this study are available upon request.
